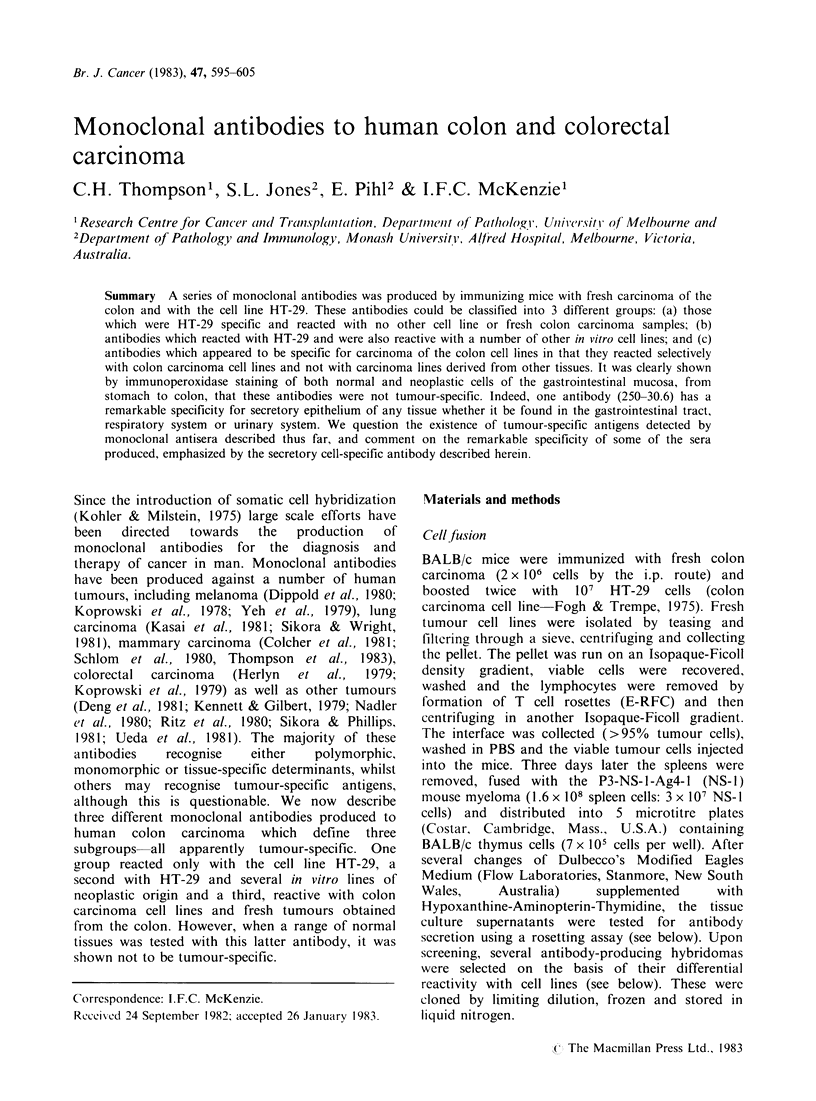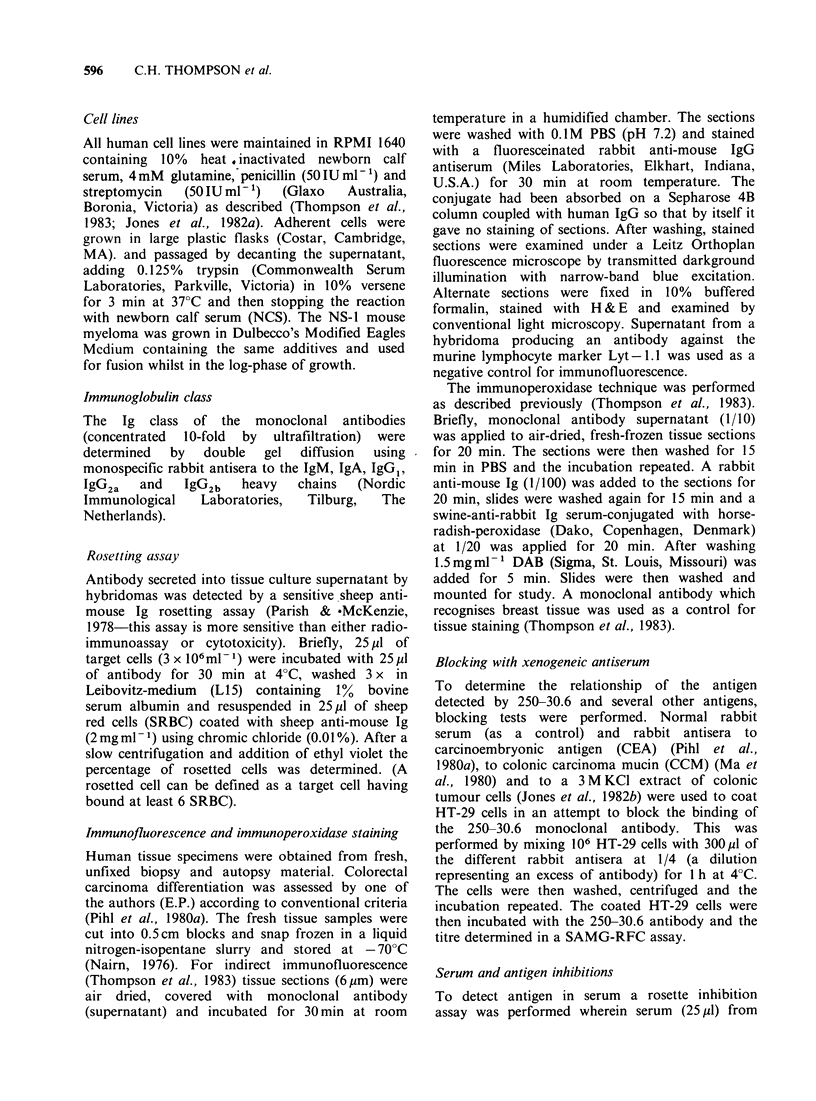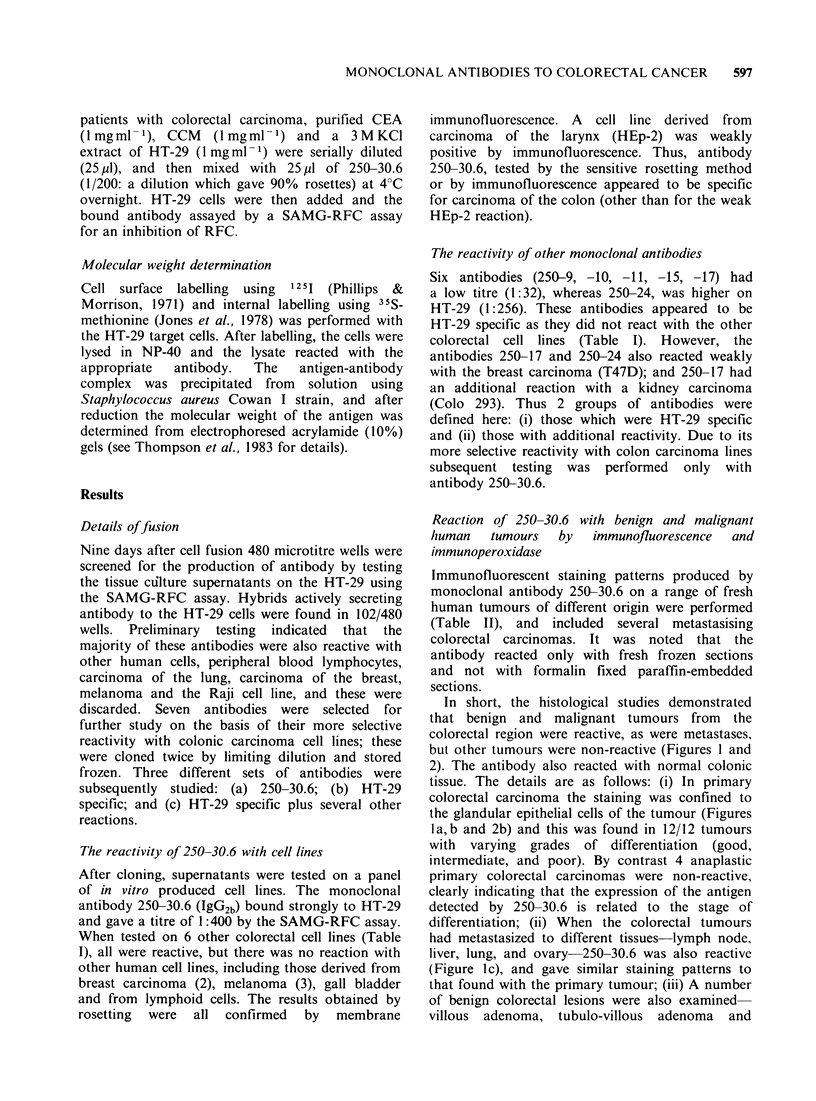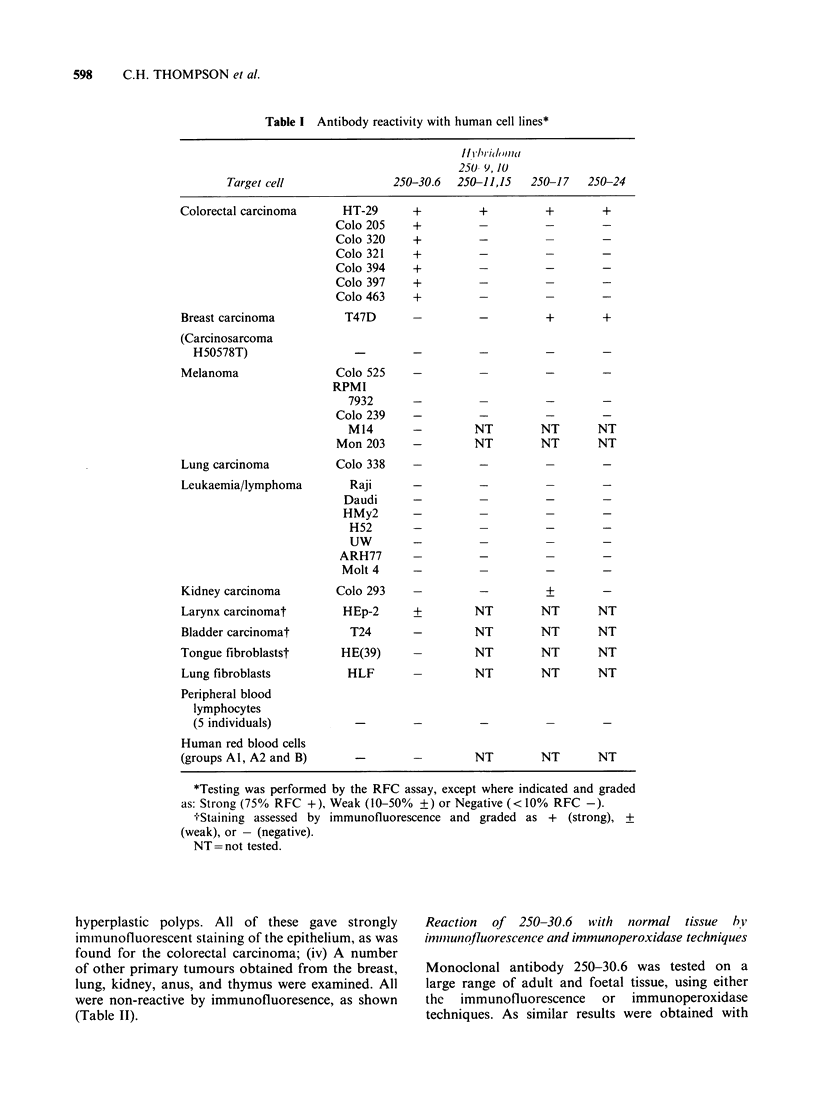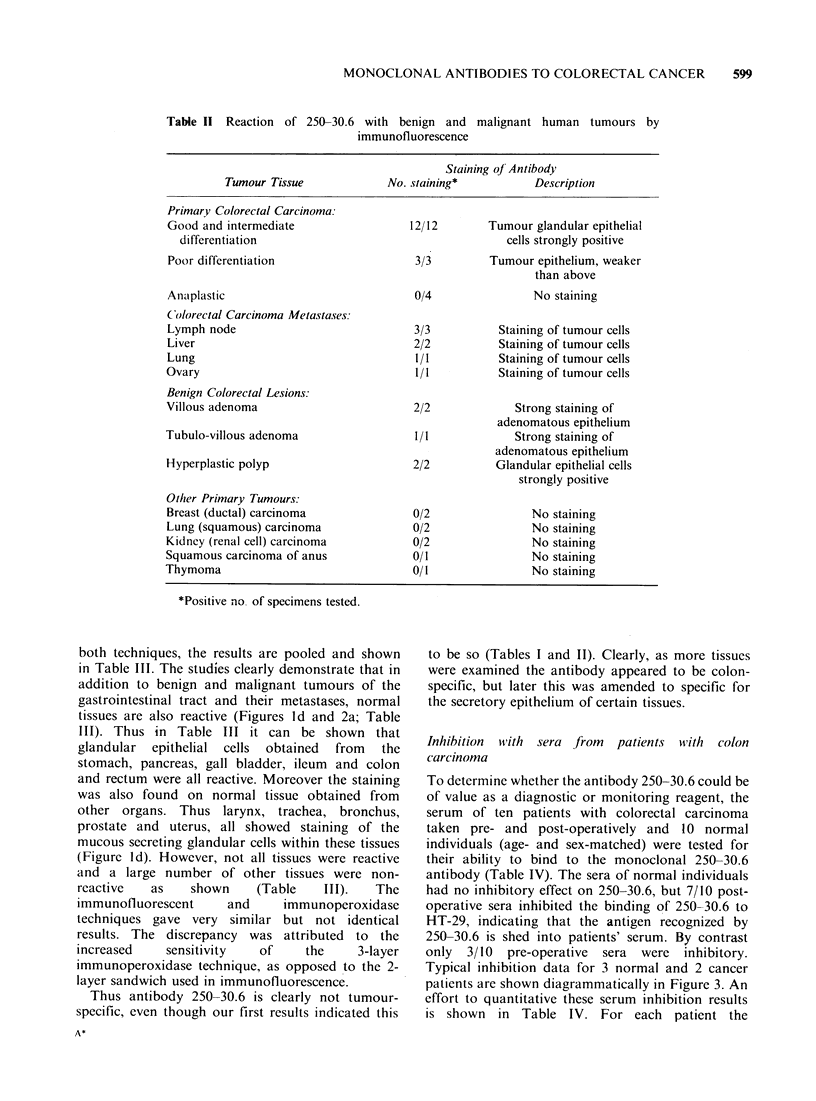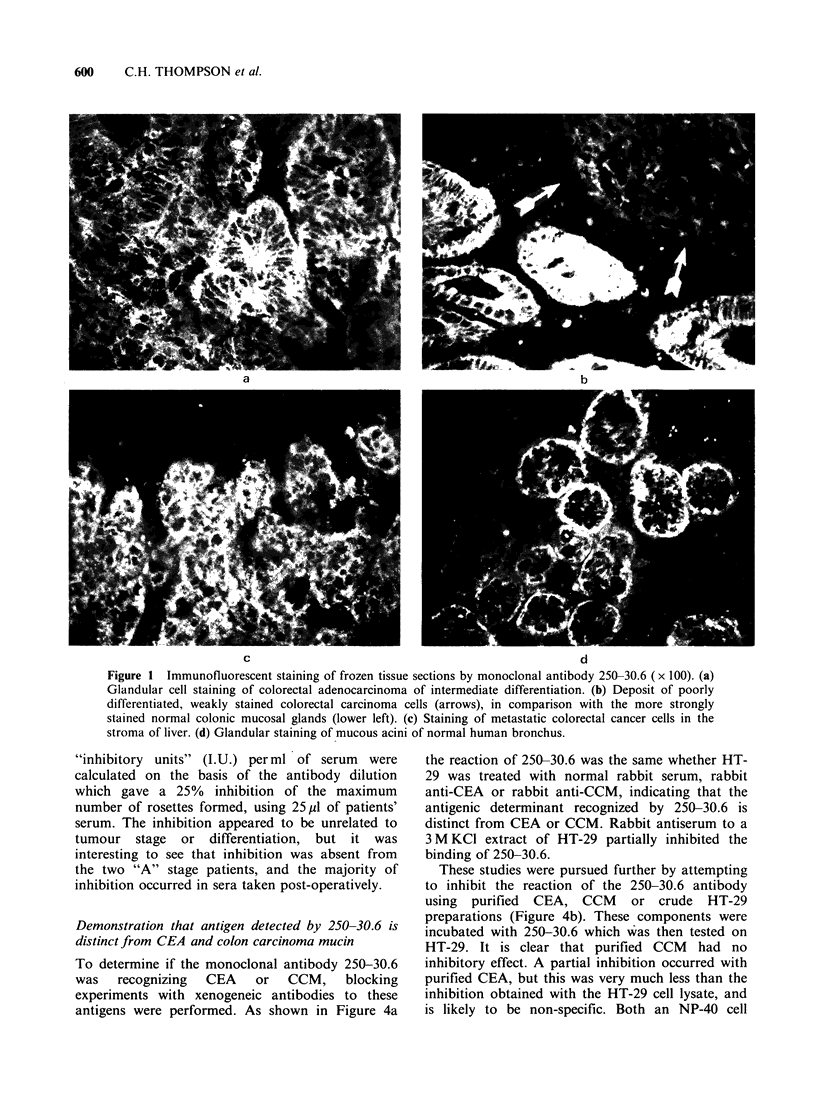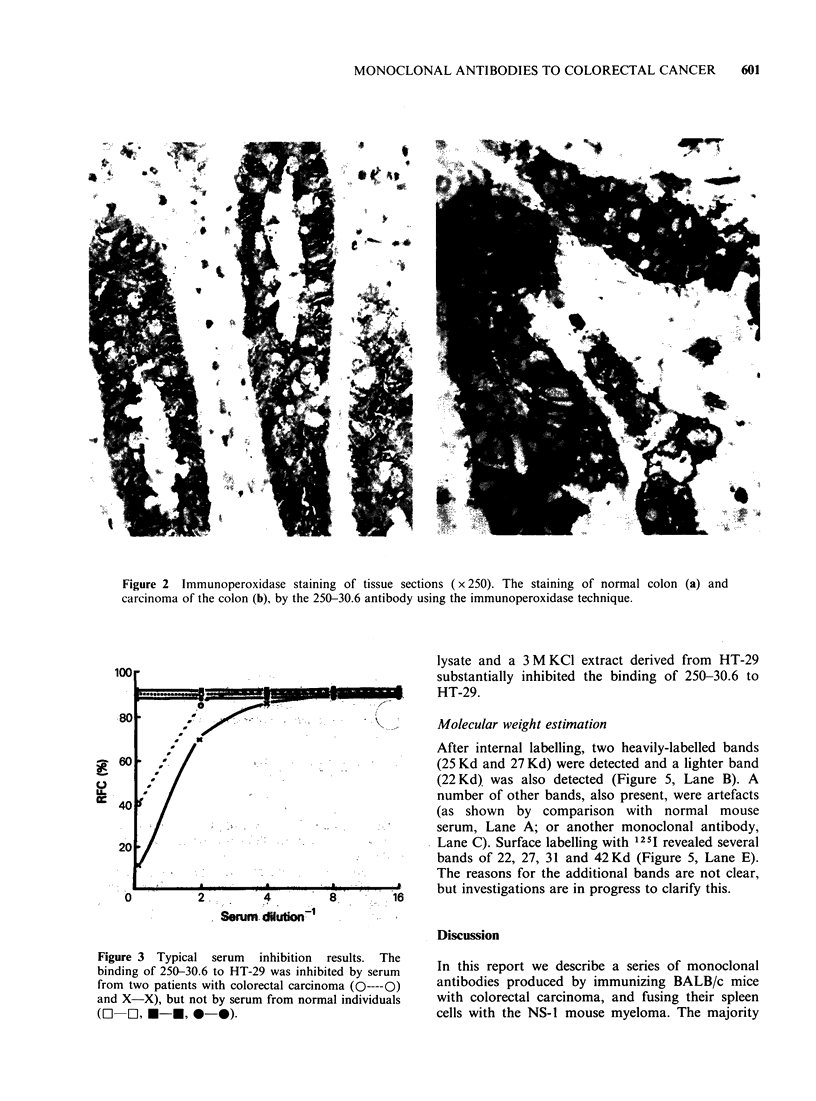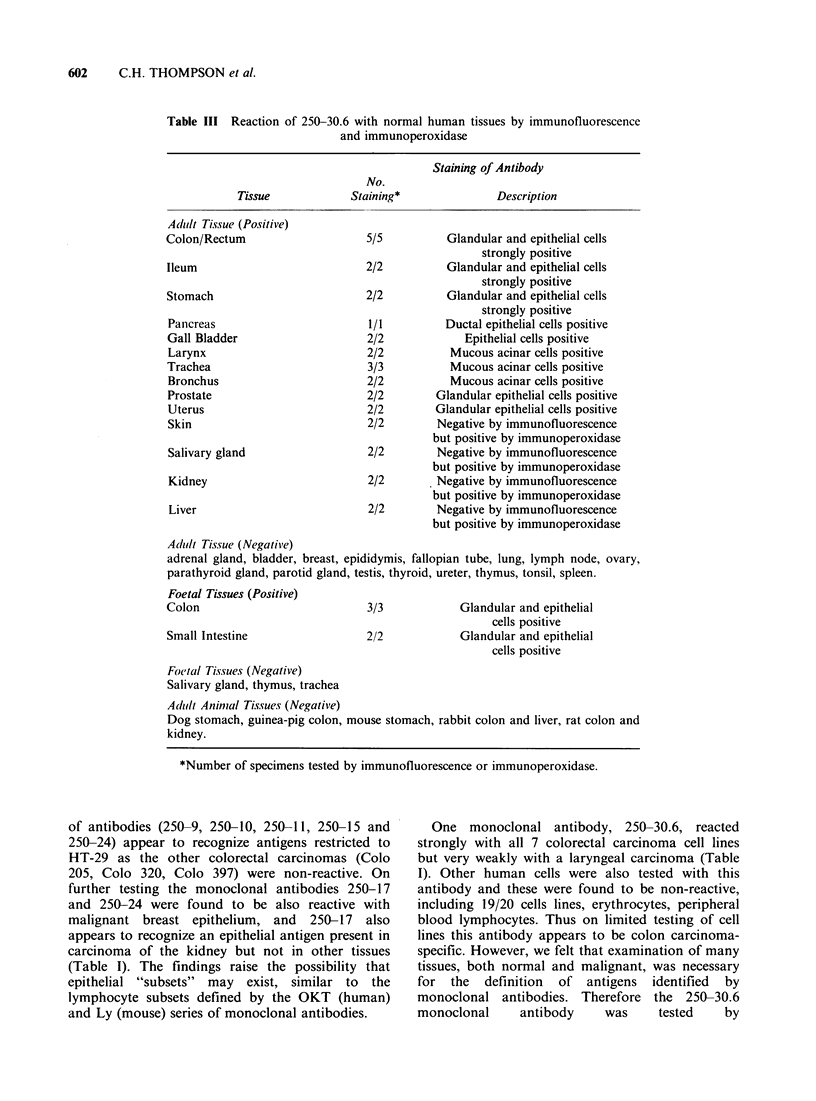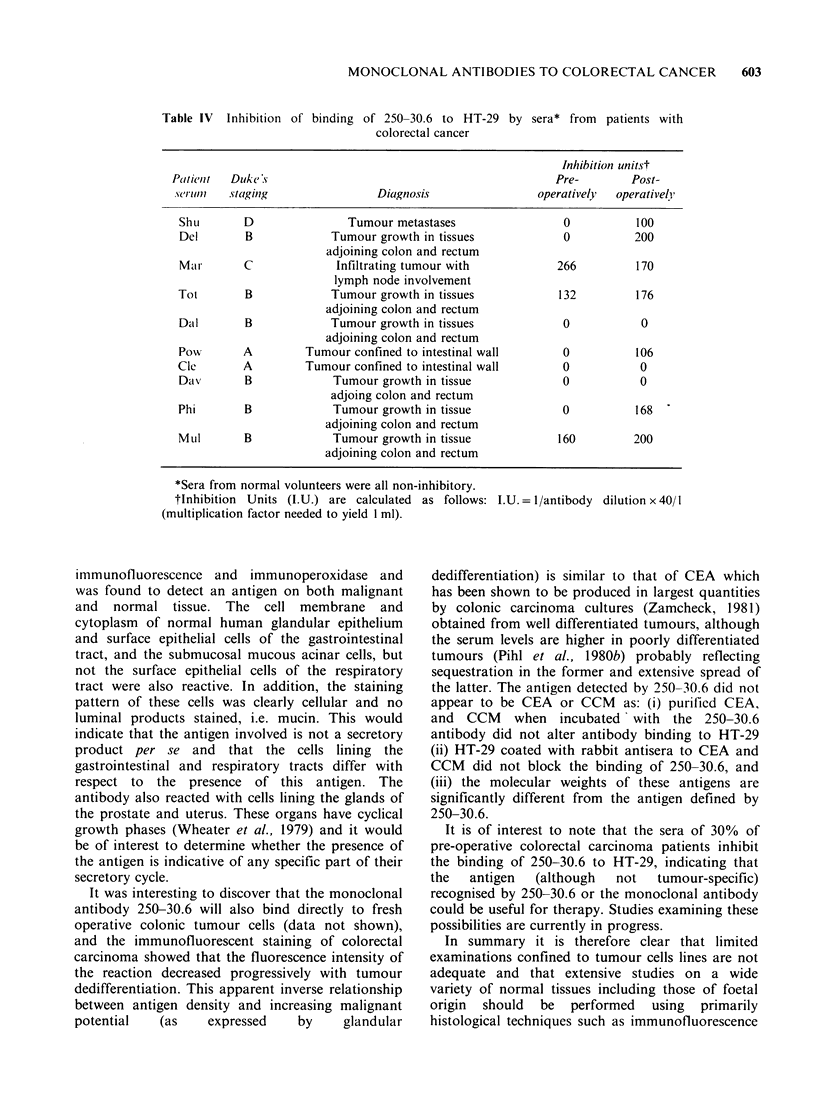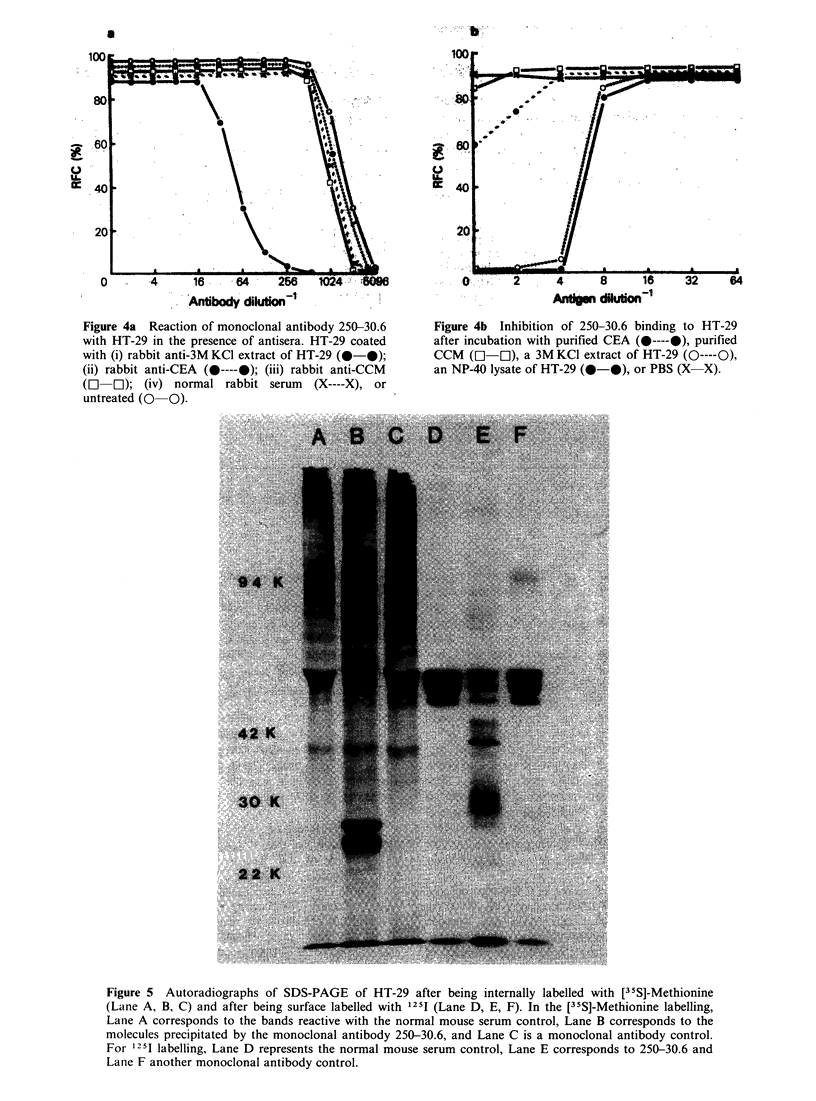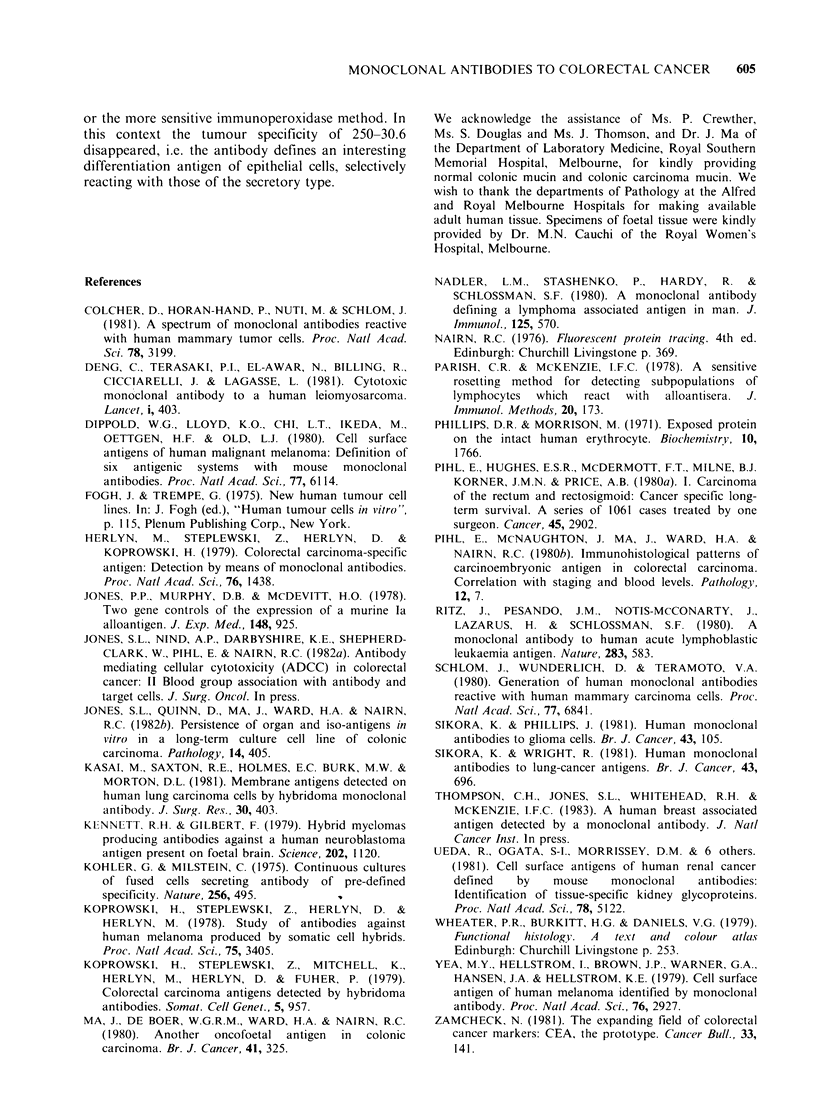# Monoclonal antibodies to human colon and colorectal carcinoma.

**DOI:** 10.1038/bjc.1983.97

**Published:** 1983-05

**Authors:** C. H. Thompson, S. L. Jones, E. Pihl, I. F. McKenzie

## Abstract

**Images:**


*Br. J. Cancer* (1983), **47**, 595–605

### Monoclonal antibodies to human colon and colorectal carcinoma

C.H. Thompson[1], S.L. Jones[2], E. Pihl[2] & I.F.C. McKenzie[1]

[1]*Research Centre for Cancer and Transplantation, Department of Pathology, University of Melbourne and* [2]*Department of Pathology and Immunology, Monash University, Alfred Hospital, Melbourne, Victoria, Australia.*

**Summary** A series of monoclonal antibodies was produced by immunizing mice with fresh carcinoma of the colon and with the cell line HT-29. These antibodies could be classified into 3 different groups: (a) those which were HT-29 specific and reacted with no other cell line or fresh colon carcinoma samples; (b) antibodies which reacted with HT-29 and were also reactive with a number of other *in vitro* cell lines; and (c) antibodies which appeared to be specific for carcinoma of the colon cell lines in that they reacted selectively with colon carcinoma cell lines and not with carcinoma lines derived from other tissues. It was clearly shown by immunoperoxidase staining of both normal and neoplastic cells of the gastrointestinal mucosa, from stomach to colon, that these antibodies were not tumour-specific. Indeed, one antibody (250–30.6) has a remarkable specificity for secretory epithelium of any tissue whether it be found in the gastrointestinal tract, respiratory system or urinary system. We question the existence of tumour-specific antigens detected by monoclonal antisera described thus far, and comment on the remarkable specificity of some of the sera produced, emphasized by the secretory cell-specific antibody described herein.

Since the introduction of somatic cell hybridization (Kohler & Milstein, 1975) large scale efforts have been directed towards the production of monoclonal antibodies for the diagnosis and therapy of cancer in man. Monoclonal antibodies have been produced against a number of human tumours, including melanoma (Dippold *et al.*, 1980; Koprowski *et al.*, 1978; Yeh *et al.*, 1979), lung carcinoma (Kasai *et al.*, 1981; Sikora & Wright, 1981), mammary carcinoma (Colcher *et al.*, 1981; Schlom *et al.*, 1980, Thompson *et al.*, 1983), colorectal carcinoma (Herlyn *et al.*, 1979; Koprowski *et al.*, 1979) as well as other tumours (Deng *et al.*, 1981; Kennett & Gilbert, 1979; Nadler *et al.*, 1980; Ritz *et al.*, 1980; Sikora & Phillips, 1981; Ueda *et al.*, 1981). The majority of these antibodies recognise either polymorphic, monomorphic or tissue-specific determinants, whilst others may recognise tumour-specific antigens, although this is questionable. We now describe three different monoclonal antibodies produced to human colon carcinoma which define three subgroups—all apparently tumour-specific. One group reacted only with the cell line HT-29, a second with HT-29 and several *in vitro* lines of neoplastic origin and a third, reactive with colon carcinoma cell lines and fresh tumours obtained from the colon. However, when a range of normal tissues was tested with this latter antibody, it was shown not to be tumour-specific.

Correspondence: I.F.C. McKenzie.

Received 24 September 1982; accepted 26 January 1983.

### Materials and methods

### *Cell fusion*

BALB/c mice were immunized with fresh colon carcinoma ($2 \times 10^6$ cells by the i.p. route) and boosted twice with $10^7$ HT-29 cells (colon carcinoma cell line—Fogh & Trempe, 1975). Fresh tumour cell lines were isolated by teasing and filtering through a sieve, centrifuging and collecting the pellet. The pellet was run on an Isopaque-Ficoll density gradient, viable cells were recovered, washed and the lymphocytes were removed by formation of T cell rosettes (E-RFC) and then centrifuging in another Isopaque-Ficoll gradient. The interface was collected (>95% tumour cells), washed in PBS and the viable tumour cells injected into the mice. Three days later the spleens were removed, fused with the P3-NS-1-Ag4-1 (NS-1) mouse myeloma ($1.6 \times 10^8$ spleen cells: $3 \times 10^7$ NS-1 cells) and distributed into 5 microtitre plates (Costar, Cambridge, Mass., U.S.A.) containing BALB/c thymus cells ($7 \times 10^5$ cells per well). After several changes of Dulbecco's Modified Eagles Medium (Flow Laboratories, Stanmore, New South Wales, Australia) supplemented with Hypoxanthine-Aminopterin-Thymidine, the tissue culture supernatants were tested for antibody secretion using a rosetting assay (see below). Upon screening, several antibody-producing hybridomas were selected on the basis of their differential reactivity with cell lines (see below). These were cloned by limiting dilution, frozen and stored in liquid nitrogen.

© The Macmillan Press Ltd., 1983

*Cell lines*

All human cell lines were maintained in RPMI 1640 containing 10% heat-inactivated newborn calf serum, 4 mM glutamine, penicillin (50 IU $ml^{-1}$) and streptomycin (50 IU $ml^{-1}$) (Glaxo Australia, Boronia, Victoria) as described (Thompson *et al.*, 1983; Jones *et al.*, 1982*a*). Adherent cells were grown in large plastic flasks (Costar, Cambridge, MA). and passaged by decanting the supernatant, adding 0.125% trypsin (Commonwealth Serum Laboratories, Parkville, Victoria) in 10% versene for 3 min at 37°C and then stopping the reaction with newborn calf serum (NCS). The NS-1 mouse myeloma was grown in Dulbecco's Modified Eagles Medium containing the same additives and used for fusion whilst in the log-phase of growth.

*Immunoglobulin class*

The Ig class of the monoclonal antibodies (concentrated 10-fold by ultrafiltration) were determined by double gel diffusion using monospecific rabbit antisera to the IgM, IgA, $IgG_1$, $IgG_{2a}$ and $IgG_{2b}$ heavy chains (Nordic Immunological Laboratories, Tilburg, The Netherlands).

*Rosetting assay*

Antibody secreted into tissue culture supernatant by hybridomas was detected by a sensitive sheep anti-mouse Ig rosetting assay (Parish & McKenzie, 1978—this assay is more sensitive than either radioimmunoassay or cytotoxicity). Briefly, 25 $\mu$l of target cells ($3 \times 10^6 ml^{-1}$) were incubated with 25 $\mu$l of antibody for 30 min at 4°C, washed 3× in Leibovitz-medium (L15) containing 1% bovine serum albumin and resuspended in 25 $\mu$l of sheep red cells (SRBC) coated with sheep anti-mouse Ig (2 mg $ml^{-1}$) using chromic chloride (0.01%). After a slow centrifugation and addition of ethyl violet the percentage of rosetted cells was determined. (A rosetted cell can be defined as a target cell having bound at least 6 SRBC).

*Immunofluorescence and immunoperoxidase staining*

Human tissue specimens were obtained from fresh, unfixed biopsy and autopsy material. Colorectal carcinoma differentiation was assessed by one of the authors (E.P.) according to conventional criteria (Pihl *et al.*, 1980*a*). The fresh tissue samples were cut into 0.5 cm blocks and snap frozen in a liquid nitrogen-isopentane slurry and stored at −70°C (Nairn, 1976). For indirect immunofluorescence (Thompson *et al.*, 1983) tissue sections (6 $\mu$m) were air dried, covered with monoclonal antibody (supernatant) and incubated for 30 min at room temperature in a humidified chamber. The sections were washed with 0.1M PBS (pH 7.2) and stained with a fluoresceinated rabbit anti-mouse IgG antiserum (Miles Laboratories, Elkhart, Indiana, U.S.A.) for 30 min at room temperature. The conjugate had been absorbed on a Sepharose 4B column coupled with human IgG so that by itself it gave no staining of sections. After washing, stained sections were examined under a Leitz Orthoplan fluorescence microscope by transmitted darkground illumination with narrow-band blue excitation. Alternate sections were fixed in 10% buffered formalin, stained with H & E and examined by conventional light microscopy. Supernatant from a hybridoma producing an antibody against the murine lymphocyte marker Lyt−1.1 was used as a negative control for immunofluorescence.

The immunoperoxidase technique was performed as described previously (Thompson *et al.*, 1983). Briefly, monoclonal antibody supernatant (1/10) was applied to air-dried, fresh-frozen tissue sections for 20 min. The sections were then washed for 15 min in PBS and the incubation repeated. A rabbit anti-mouse Ig (1/100) was added to the sections for 20 min, slides were washed again for 15 min and a swine-anti-rabbit Ig serum-conjugated with horse-radish-peroxidase (Dako, Copenhagen, Denmark) at 1/20 was applied for 20 min. After washing 1.5 mg $ml^{-1}$ DAB (Sigma, St. Louis, Missouri) was added for 5 min. Slides were then washed and mounted for study. A monoclonal antibody which recognises breast tissue was used as a control for tissue staining (Thompson *et al.*, 1983).

*Blocking with xenogeneic antiserum*

To determine the relationship of the antigen detected by 250–30.6 and several other antigens, blocking tests were performed. Normal rabbit serum (as a control) and rabbit antisera to carcinoembryonic antigen (CEA) (Pihl *et al.*, 1980*a*), to colonic carcinoma mucin (CCM) (Ma *et al.*, 1980) and to a 3 M KCl extract of colonic tumour cells (Jones *et al.*, 1982*b*) were used to coat HT-29 cells in an attempt to block the binding of the 250–30.6 monoclonal antibody. This was performed by mixing $10^6$ HT-29 cells with 300 $\mu$l of the different rabbit antisera at 1/4 (a dilution representing an excess of antibody) for 1 h at 4°C. The cells were then washed, centrifuged and the incubation repeated. The coated HT-29 cells were then incubated with the 250–30.6 antibody and the titre determined in a SAMG-RFC assay.

*Serum and antigen inhibitions*

To detect antigen in serum a rosette inhibition assay was performed wherein serum (25 $\mu$l) from patients with colorectal carcinoma, purified CEA ($1\,mg\,ml^{-1}$), CCM ($1\,mg\,ml^{-1}$) and a 3 M KCl extract of HT-29 ($1\,mg\,ml^{-1}$) were serially diluted ($25\,\mu l$), and then mixed with $25\,\mu l$ of 250–30.6 (1/200: a dilution which gave 90% rosettes) at 4°C overnight. HT-29 cells were then added and the bound antibody assayed by a SAMG-RFC assay for an inhibition of RFC.

*Molecular weight determination*

Cell surface labelling using $^{125}I$ (Phillips & Morrison, 1971) and internal labelling using $^{35}S$-methionine (Jones *et al.*, 1978) was performed with the HT-29 target cells. After labelling, the cells were lysed in NP-40 and the lysate reacted with the appropriate antibody. The antigen-antibody complex was precipitated from solution using *Staphylococcus aureus* Cowan I strain, and after reduction the molecular weight of the antigen was determined from electrophoresed acrylamide (10%) gels (see Thompson *et al.*, 1983 for details).

### Results

*Details of fusion*

Nine days after cell fusion 480 microtitre wells were screened for the production of antibody by testing the tissue culture supernatants on the HT-29 using the SAMG-RFC assay. Hybrids actively secreting antibody to the HT-29 cells were found in 102/480 wells. Preliminary testing indicated that the majority of these antibodies were also reactive with other human cells, peripheral blood lymphocytes, carcinoma of the lung, carcinoma of the breast, melanoma and the Raji cell line, and these were discarded. Seven antibodies were selected for further study on the basis of their more selective reactivity with colonic carcinoma cell lines; these were cloned twice by limiting dilution and stored frozen. Three different sets of antibodies were subsequently studied: (a) 250–30.6; (b) HT-29 specific; and (c) HT-29 specific plus several other reactions.

*The reactivity of 250–30.6 with cell lines*

After cloning, supernatants were tested on a panel of *in vitro* produced cell lines. The monoclonal antibody 250–30.6 ($IgG_{2b}$) bound strongly to HT-29 and gave a titre of 1:400 by the SAMG-RFC assay. When tested on 6 other colorectal cell lines (Table I), all were reactive, but there was no reaction with other human cell lines, including those derived from breast carcinoma (2), melanoma (3), gall bladder and from lymphoid cells. The results obtained by rosetting were all confirmed by membrane immunofluorescence. A cell line derived from carcinoma of the larynx (HEp-2) was weakly positive by immunofluorescence. Thus, antibody 250–30.6, tested by the sensitive rosetting method or by immunofluorescence appeared to be specific for carcinoma of the colon (other than for the weak HEp-2 reaction).

*The reactivity of other monoclonal antibodies*

Six antibodies (250–9, –10, –11, –15, –17) had a low titre (1:32), whereas 250–24, was higher on HT-29 (1:256). These antibodies appeared to be HT-29 specific as they did not react with the other colorectal cell lines (Table I). However, the antibodies 250–17 and 250–24 also reacted weakly with the breast carcinoma (T47D); and 250–17 had an additional reaction with a kidney carcinoma (Colo 293). Thus 2 groups of antibodies were defined here: (i) those which were HT-29 specific and (ii) those with additional reactivity. Due to its more selective reactivity with colon carcinoma lines subsequent testing was performed only with antibody 250–30.6.

*Reaction of 250–30.6 with benign and malignant human tumours by immunofluorescence and immunoperoxidase*

Immunofluorescent staining patterns produced by monoclonal antibody 250–30.6 on a range of fresh human tumours of different origin were performed (Table II), and included several metastasising colorectal carcinomas. It was noted that the antibody reacted only with fresh frozen sections and not with formalin fixed paraffin-embedded sections.

In short, the histological studies demonstrated that benign and malignant tumours from the colorectal region were reactive, as were metastases, but other tumours were non-reactive (Figures 1 and 2). The antibody also reacted with normal colonic tissue. The details are as follows: (i) In primary colorectal carcinoma the staining was confined to the glandular epithelial cells of the tumour (Figures 1a, b and 2b) and this was found in 12/12 tumours with varying grades of differentiation (good, intermediate, and poor). By contrast 4 anaplastic primary colorectal carcinomas were non-reactive, clearly indicating that the expression of the antigen detected by 250–30.6 is related to the stage of differentiation; (ii) When the colorectal tumours had metastasized to different tissues—lymph node, liver, lung, and ovary—250–30.6 was also reactive (Figure 1c), and gave similar staining patterns to that found with the primary tumour; (iii) A number of benign colorectal lesions were also examined—villous adenoma, tubulo-villous adenoma and

**Table I** Antibody reactivity with human cell lines*

| *Target cell* | | | *Hybridoma* | | |
|---|---|---|---|---|---|
| | | *250–30.6* | *250–9,10 250–11,15* | *250–17* | *250–24* |
| Colorectal carcinoma | HT-29 | + | + | + | + |
| | Colo 205 | + | – | – | – |
| | Colo 320 | + | – | – | – |
| | Colo 321 | + | – | – | – |
| | Colo 394 | + | – | – | – |
| | Colo 397 | + | – | – | – |
| | Colo 463 | + | – | – | – |
| Breast carcinoma | T47D | – | – | + | + |
| (Carcinosarcoma H50578T) | — | – | – | – | – |
| Melanoma | Colo 525 | – | – | – | – |
| | RPMI 7932 | – | – | – | – |
| | Colo 239 | – | – | – | – |
| | M14 | – | NT | NT | NT |
| | Mon 203 | – | NT | NT | NT |
| Lung carcinoma | Colo 338 | – | – | – | – |
| Leukaemia/lymphoma | Raji | – | – | – | – |
| | Daudi | – | – | – | – |
| | HMy2 | – | – | – | – |
| | H52 | – | – | – | – |
| | UW | – | – | – | – |
| | ARH77 | – | – | – | – |
| | Molt 4 | – | – | – | – |
| Kidney carcinoma | Colo 293 | – | – | ± | – |
| Larynx carcinoma† | HEp-2 | ± | NT | NT | NT |
| Bladder carcinoma† | T24 | – | NT | NT | NT |
| Tongue fibroblasts† | HE(39) | – | NT | NT | NT |
| Lung fibroblasts | HLF | – | NT | NT | NT |
| Peripheral blood lymphocytes (5 individuals) | — | – | – | – | – |
| Human red blood cells (groups A1, A2 and B) | — | – | NT | NT | NT |

*Testing was performed by the RFC assay, except where indicated and graded as: Strong (75% RFC +), Weak (10–50% ±) or Negative (<10% RFC –).
†Staining assessed by immunofluorescence and graded as + (strong), ± (weak), or – (negative).
NT = not tested.

hyperplastic polyps. All of these gave strongly immunofluorescent staining of the epithelium, as was found for the colorectal carcinoma; (iv) A number of other primary tumours obtained from the breast, lung, kidney, anus, and thymus were examined. All were non-reactive by immunofluoresence, as shown (Table II).

*Reaction of 250–30.6 with normal tissue by immunofluorescence and immunoperoxidase techniques*

Monoclonal antibody 250–30.6 was tested on a large range of adult and foetal tissue, using either the immunofluorescence or immunoperoxidase techniques. As similar results were obtained with

**Table II** Reaction of 250–30.6 with benign and malignant human tumours by immunofluorescence

| Tumour Tissue | Staining of Antibody No. staining* | Description |
|---|---|---|
| *Primary Colorectal Carcinoma:* | | |
| Good and intermediate differentiation | 12/12 | Tumour glandular epithelial cells strongly positive |
| Poor differentiation | 3/3 | Tumour epithelium, weaker than above |
| Anaplastic | 0/4 | No staining |
| *Colorectal Carcinoma Metastases:* | | |
| Lymph node | 3/3 | Staining of tumour cells |
| Liver | 2/2 | Staining of tumour cells |
| Lung | 1/1 | Staining of tumour cells |
| Ovary | 1/1 | Staining of tumour cells |
| *Benign Colorectal Lesions:* | | |
| Villous adenoma | 2/2 | Strong staining of adenomatous epithelium |
| Tubulo-villous adenoma | 1/1 | Strong staining of adenomatous epithelium |
| Hyperplastic polyp | 2/2 | Glandular epithelial cells strongly positive |
| *Other Primary Tumours:* | | |
| Breast (ductal) carcinoma | 0/2 | No staining |
| Lung (squamous) carcinoma | 0/2 | No staining |
| Kidney (renal cell) carcinoma | 0/2 | No staining |
| Squamous carcinoma of anus | 0/1 | No staining |
| Thymoma | 0/1 | No staining |

*Positive no. of specimens tested.

both techniques, the results are pooled and shown in Table III. The studies clearly demonstrate that in addition to benign and malignant tumours of the gastrointestinal tract and their metastases, normal tissues are also reactive (Figures 1d and 2a; Table III). Thus in Table III it can be shown that glandular epithelial cells obtained from the stomach, pancreas, gall bladder, ileum and colon and rectum were all reactive. Moreover the staining was also found on normal tissue obtained from other organs. Thus larynx, trachea, bronchus, prostate and uterus, all showed staining of the mucous secreting glandular cells within these tissues (Figure 1d). However, not all tissues were reactive and a large number of other tissues were non-reactive as shown (Table III). The immunofluorescent and immunoperoxidase techniques gave very similar but not identical results. The discrepancy was attributed to the increased sensitivity of the 3-layer immunoperoxidase technique, as opposed to the 2-layer sandwich used in immunofluorescence.

Thus antibody 250–30.6 is clearly not tumour-specific, even though our first results indicated this to be so (Tables I and II). Clearly, as more tissues were examined the antibody appeared to be colon-specific, but later this was amended to specific for the secretory epithelium of certain tissues.

*Inhibition with sera from patients with colon carcinoma*

To determine whether the antibody 250–30.6 could be of value as a diagnostic or monitoring reagent, the serum of ten patients with colorectal carcinoma taken pre- and post-operatively and 10 normal individuals (age- and sex-matched) were tested for their ability to bind to the monoclonal 250–30.6 antibody (Table IV). The sera of normal individuals had no inhibitory effect on 250–30.6, but 7/10 post-operative sera inhibited the binding of 250–30.6 to HT-29, indicating that the antigen recognized by 250–30.6 is shed into patients' serum. By contrast only 3/10 pre-operative sera were inhibitory. Typical inhibition data for 3 normal and 2 cancer patients are shown diagrammatically in Figure 3. An effort to quantitative these serum inhibition results is shown in Table IV. For each patient the

**Figure 1** Immunofluorescent staining of frozen tissue sections by monoclonal antibody 250–30.6 (×100). (**a**) Glandular cell staining of colorectal adenocarcinoma of intermediate differentiation. (**b**) Deposit of poorly differentiated, weakly stained colorectal carcinoma cells (arrows), in comparison with the more strongly stained normal colonic mucosal glands (lower left). (**c**) Staining of metastatic colorectal cancer cells in the stroma of liver. (**d**) Glandular staining of mucous acini of normal human bronchus.

"inhibitory units" (I.U.) per ml of serum were calculated on the basis of the antibody dilution which gave a 25% inhibition of the maximum number of rosettes formed, using 25 $\mu$l of patients' serum. The inhibition appeared to be unrelated to tumour stage or differentiation, but it was interesting to see that inhibition was absent from the two "A" stage patients, and the majority of inhibition occurred in sera taken post-operatively.

*Demonstration that antigen detected by 250–30.6 is distinct from CEA and colon carcinoma mucin*

To determine if the monoclonal antibody 250–30.6 was recognizing CEA or CCM, blocking experiments with xenogeneic antibodies to these antigens were performed. As shown in Figure 4a the reaction of 250–30.6 was the same whether HT-29 was treated with normal rabbit serum, rabbit anti-CEA or rabbit anti-CCM, indicating that the antigenic determinant recognized by 250–30.6 is distinct from CEA or CCM. Rabbit antiserum to a 3 M KCl extract of HT-29 partially inhibited the binding of 250–30.6.

These studies were pursued further by attempting to inhibit the reaction of the 250–30.6 antibody using purified CEA, CCM or crude HT-29 preparations (Figure 4b). These components were incubated with 250–30.6 which was then tested on HT-29. It is clear that purified CCM had no inhibitory effect. A partial inhibition occurred with purified CEA, but this was very much less than the inhibition obtained with the HT-29 cell lysate, and is likely to be non-specific. Both an NP-40 cell

**Figure 2** Immunoperoxidase staining of tissue sections (×250). The staining of normal colon (**a**) and carcinoma of the colon (**b**), by the 250–30.6 antibody using the immunoperoxidase technique.

**Figure 3** Typical serum inhibition results. The binding of 250–30.6 to HT-29 was inhibited by serum from two patients with colorectal carcinoma (○----○) and X—X), but not by serum from normal individuals (□—□, ■—■, ●—●).

lysate and a 3 M KCl extract derived from HT-29 substantially inhibited the binding of 250–30.6 to HT-29.

### *Molecular weight estimation*

After internal labelling, two heavily-labelled bands (25 Kd and 27 Kd) were detected and a lighter band (22 Kd) was also detected (Figure 5, Lane B). A number of other bands, also present, were artefacts (as shown by comparison with normal mouse serum, Lane A; or another monoclonal antibody, Lane C). Surface labelling with $^{125}I$ revealed several bands of 22, 27, 31 and 42 Kd (Figure 5, Lane E). The reasons for the additional bands are not clear, but investigations are in progress to clarify this.

### Discussion

In this report we describe a series of monoclonal antibodies produced by immunizing BALB/c mice with colorectal carcinoma, and fusing their spleen cells with the NS-1 mouse myeloma. The majority

**Table III** Reaction of 250–30.6 with normal human tissues by immunofluorescence and immunoperoxidase

| Tissue | No. Staining* | Staining of Antibody: Description |
|---|---|---|
| *Adult Tissue (Positive)* | | |
| Colon/Rectum | 5/5 | Glandular and epithelial cells strongly positive |
| Ileum | 2/2 | Glandular and epithelial cells strongly positive |
| Stomach | 2/2 | Glandular and epithelial cells strongly positive |
| Pancreas | 1/1 | Ductal epithelial cells positive |
| Gall Bladder | 2/2 | Epithelial cells positive |
| Larynx | 2/2 | Mucous acinar cells positive |
| Trachea | 3/3 | Mucous acinar cells positive |
| Bronchus | 2/2 | Mucous acinar cells positive |
| Prostate | 2/2 | Glandular epithelial cells positive |
| Uterus | 2/2 | Glandular epithelial cells positive |
| Skin | 2/2 | Negative by immunofluorescence but positive by immunoperoxidase |
| Salivary gland | 2/2 | Negative by immunofluorescence but positive by immunoperoxidase |
| Kidney | 2/2 | Negative by immunofluorescence but positive by immunoperoxidase |
| Liver | 2/2 | Negative by immunofluorescence but positive by immunoperoxidase |
| *Adult Tissue (Negative)* | | |
| adrenal gland, bladder, breast, epididymis, fallopian tube, lung, lymph node, ovary, parathyroid gland, parotid gland, testis, thyroid, ureter, thymus, tonsil, spleen. | | |
| ***Foetal Tissues (Positive)*** | | |
| Colon | 3/3 | Glandular and epithelial cells positive |
| Small Intestine | 2/2 | Glandular and epithelial cells positive |
| *Foetal Tissues (Negative)* | | |
| Salivary gland, thymus, trachea | | |
| *Adult Animal Tissues (Negative)* | | |
| Dog stomach, guinea-pig colon, mouse stomach, rabbit colon and liver, rat colon and kidney. | | |

*Number of specimens tested by immunofluorescence or immunoperoxidase.

of antibodies (250–9, 250–10, 250–11, 250–15 and 250–24) appear to recognize antigens restricted to HT-29 as the other colorectal carcinomas (Colo 205, Colo 320, Colo 397) were non-reactive. On further testing the monoclonal antibodies 250–17 and 250–24 were found to be also reactive with malignant breast epithelium, and 250–17 also appears to recognize an epithelial antigen present in carcinoma of the kidney but not in other tissues (Table I). The findings raise the possibility that epithelial "subsets" may exist, similar to the lymphocyte subsets defined by the OKT (human) and Ly (mouse) series of monoclonal antibodies.

One monoclonal antibody, 250–30.6, reacted strongly with all 7 colorectal carcinoma cell lines but very weakly with a laryngeal carcinoma (Table I). Other human cells were also tested with this antibody and these were found to be non-reactive, including 19/20 cells lines, erythrocytes, peripheral blood lymphocytes. Thus on limited testing of cell lines this antibody appears to be colon carcinoma-specific. However, we felt that examination of many tissues, both normal and malignant, was necessary for the definition of antigens identified by monoclonal antibodies. Therefore the 250–30.6 monoclonal antibody was tested by

**Table IV** Inhibition of binding of 250–30.6 to HT-29 by sera* from patients with colorectal cancer

| *Patient serum* | *Duke's staging* | *Diagnosis* | *Inhibition units†* *Pre-operatively* | *Post-operatively* |
|---|---|---|---|---|
| Shu | D | Tumour metastases | 0 | 100 |
| Del | B | Tumour growth in tissues adjoining colon and rectum | 0 | 200 |
| Mar | C | Infiltrating tumour with lymph node involvement | 266 | 170 |
| Tot | B | Tumour growth in tissues adjoining colon and rectum | 132 | 176 |
| Dal | B | Tumour growth in tissues adjoining colon and rectum | 0 | 0 |
| Pow | A | Tumour confined to intestinal wall | 0 | 106 |
| Cle | A | Tumour confined to intestinal wall | 0 | 0 |
| Dav | B | Tumour growth in tissue adjoing colon and rectum | 0 | 0 |
| Phi | B | Tumour growth in tissue adjoining colon and rectum | 0 | 168 |
| Mul | B | Tumour growth in tissue adjoining colon and rectum | 160 | 200 |

*Sera from normal volunteers were all non-inhibitory.

†Inhibition Units (I.U.) are calculated as follows: I.U. = 1/antibody dilution × 40/1 (multiplication factor needed to yield 1 ml).

immunofluorescence and immunoperoxidase and was found to detect an antigen on both malignant and normal tissue. The cell membrane and cytoplasm of normal human glandular epithelium and surface epithelial cells of the gastrointestinal tract, and the submucosal mucous acinar cells, but not the surface epithelial cells of the respiratory tract were also reactive. In addition, the staining pattern of these cells was clearly cellular and no luminal products stained, i.e. mucin. This would indicate that the antigen involved is not a secretory product *per se* and that the cells lining the gastrointestinal and respiratory tracts differ with respect to the presence of this antigen. The antibody also reacted with cells lining the glands of the prostate and uterus. These organs have cyclical growth phases (Wheater *et al.*, 1979) and it would be of interest to determine whether the presence of the antigen is indicative of any specific part of their secretory cycle.

It was interesting to discover that the monoclonal antibody 250–30.6 will also bind directly to fresh operative colonic tumour cells (data not shown), and the immunofluorescent staining of colorectal carcinoma showed that the fluorescence intensity of the reaction decreased progressively with tumour dedifferentiation. This apparent inverse relationship between antigen density and increasing malignant potential (as expressed by glandular dedifferentiation) is similar to that of CEA which has been shown to be produced in largest quantities by colonic carcinoma cultures (Zamcheck, 1981) obtained from well differentiated tumours, although the serum levels are higher in poorly differentiated tumours (Pihl *et al.*, 1980*b*) probably reflecting sequestration in the former and extensive spread of the latter. The antigen detected by 250–30.6 did not appear to be CEA or CCM as: (i) purified CEA, and CCM when incubated with the 250–30.6 antibody did not alter antibody binding to HT-29 (ii) HT-29 coated with rabbit antisera to CEA and CCM did not block the binding of 250–30.6, and (iii) the molecular weights of these antigens are significantly different from the antigen defined by 250–30.6.

It is of interest to note that the sera of 30% of pre-operative colorectal carcinoma patients inhibit the binding of 250–30.6 to HT-29, indicating that the antigen (although not tumour-specific) recognised by 250–30.6 or the monoclonal antibody could be useful for therapy. Studies examining these possibilities are currently in progress.

In summary it is therefore clear that limited examinations confined to tumour cells lines are not adequate and that extensive studies on a wide variety of normal tissues including those of foetal origin should be performed using primarily histological techniques such as immunofluorescence

**Figure 4a** Reaction of monoclonal antibody 250–30.6 with HT-29 in the presence of antisera. HT-29 coated with (i) rabbit anti-3M KCl extract of HT-29 (●—●); (ii) rabbit anti-CEA (●----●); (iii) rabbit anti-CCM (□—□); (iv) normal rabbit serum (X----X), or untreated (○—○).

**Figure 4b** Inhibition of 250–30.6 binding to HT-29 after incubation with purified CEA (●----●), purified CCM (□—□), a 3M KCl extract of HT-29 (○----○), an NP-40 lysate of HT-29 (●—●), or PBS (X—X).

**Figure 5** Autoradiographs of SDS-PAGE of HT-29 after being internally labelled with [$^{35}$S]-Methionine (Lane A, B, C) and after being surface labelled with $^{125}$I (Lane D, E, F). In the [$^{35}$S]-Methionine labelling, Lane A corresponds to the bands reactive with the normal mouse serum control, Lane B corresponds to the molecules precipitated by the monoclonal antibody 250–30.6, and Lane C is a monoclonal antibody control. For $^{125}$I labelling, Lane D represents the normal mouse serum control, Lane E corresponds to 250–30.6 and Lane F another monoclonal antibody control.

or the more sensitive immunoperoxidase method. In this context the tumour specificity of 250–30.6 disappeared, i.e. the antibody defines an interesting differentiation antigen of epithelial cells, selectively reacting with those of the secretory type.

We acknowledge the assistance of Ms. P. Crewther, Ms. S. Douglas and Ms. J. Thomson, and Dr. J. Ma of the Department of Laboratory Medicine, Royal Southern Memorial Hospital, Melbourne, for kindly providing normal colonic mucin and colonic carcinoma mucin. We wish to thank the departments of Pathology at the Alfred and Royal Melbourne Hospitals for making available adult human tissue. Specimens of foetal tissue were kindly provided by Dr. M.N. Cauchi of the Royal Women's Hospital, Melbourne.

### References

COLCHER, D., HORAN-HAND, P., NUTI, M. & SCHLOM, J. (1981). A spectrum of monoclonal antibodies reactive with human mammary tumor cells. *Proc. Natl Acad. Sci.* **78,** 3199.

DENG, C., TERASAKI, P.I., EL-AWAR, N., BILLING, R., CICCIARELLI, J. & LAGASSE, L. (1981). Cytotoxic monoclonal antibody to a human leiomyosarcoma. *Lancet,* **i,** 403.

DIPPOLD, W.G., LLOYD, K.O., CHI, L.T., IKEDA, M., OETTGEN, H.F. & OLD, L.J. (1980). Cell surface antigens of human malignant melanoma: Definition of six antigenic systems with mouse monoclonal antibodies. *Proc. Natl Acad. Sci.,* **77,** 6114.

FOGH, J. & TREMPE, G. (1975). New human tumour cell lines. In: J. Fogh (ed.), "Human tumour cells *in vitro*", p. 115, Plenum Publishing Corp., New York.

HERLYN, M., STEPLEWSKI, Z., HERLYN, D. & KOPROWSKI, H. (1979). Colorectal carcinoma-specific antigen: Detection by means of monoclonal antibodies. *Proc. Natl Acad. Sci.,* **76,** 1438.

JONES, P.P., MURPHY, D.B. & MCDEVITT, H.O. (1978). Two gene controls of the expression of a murine Ia alloantigen. *J. Exp. Med.,* **148,** 925.

JONES, S.L., NIND, A.P., DARBYSHIRE, K.E., SHEPHERD-CLARK, W., PIHL, E. & NAIRN, R.C. (1982*a*). Antibody mediating cellular cytotoxicity (ADCC) in colorectal cancer: II Blood group association with antibody and target cells. *J. Surg. Oncol.* In press.

JONES, S.L., QUINN, D., MA, J., WARD, H.A. & NAIRN, R.C. (1982*b*). Persistence of organ and iso-antigens *in vitro* in a long-term culture cell line of colonic carcinoma. *Pathology,* **14,** 405.

KASAI, M., SAXTON, R.E., HOLMES, E.C. BURK, M.W. & MORTON, D.L. (1981). Membrane antigens detected on human lung carcinoma cells by hybridoma monoclonal antibody. *J. Surg. Res.,* **30,** 403.

KENNETT, R.H. & GILBERT, F. (1979). Hybrid myelomas producing antibodies against a human neuroblastoma antigen present on foetal brain. *Science,* **202,** 1120.

KOHLER, G. & MILSTEIN, C. (1975). Continuous cultures of fused cells secreting antibody of pre-defined specificity. *Nature,* **256,** 495.

KOPROWSKI, H., STEPLEWSKI, Z., HERLYN, D. & HERLYN, M. (1978). Study of antibodies against human melanoma produced by somatic cell hybrids. *Proc. Natl Acad. Sci.,* **75,** 3405.

KOPROWSKI, H., STEPLEWSKI, Z., MITCHELL, K., HERLYN, M., HERLYN, D. & FUHER, P. (1979). Colorectal carcinoma antigens detected by hybridoma antibodies. *Somat. Cell Genet.,* **5,** 957.

MA, J., DE BOER, W.G.R.M., WARD, H.A. & NAIRN, R.C. (1980). Another oncofoetal antigen in colonic carcinoma. *Br. J. Cancer,* **41,** 325.

NADLER, L.M., STASHENKO, P., HARDY, R. & SCHLOSSMAN, S.F. (1980). A monoclonal antibody defining a lymphoma associated antigen in man. *J. Immunol.,* **125,** 570.

NAIRN, R.C. (1976). *Fluorescent protein tracing.* 4th ed. Edinburgh: Churchill Livingstone p. 369.

PARISH, C.R. & MCKENZIE, I.F.C. (1978). A sensitive rosetting method for detecting subpopulations of lymphocytes which react with alloantisera. *J. Immunol. Methods,* **20,** 173.

PHILLIPS, D.R. & MORRISON, M. (1971). Exposed protein on the intact human erythrocyte. *Biochemistry,* **10,** 1766.

PIHL, E., HUGHES, E.S.R., MCDERMOTT, F.T., MILNE, B.J. KORNER, J.M.N. & PRICE, A.B. (1980*a*). I. Carcinoma of the rectum and rectosigmoid: Cancer specific long-term survival. A series of 1061 cases treated by one surgeon. *Cancer,* **45,** 2902.

PIHL, E., MCNAUGHTON, J. MA, J., WARD, H.A. & NAIRN, R.C. (1980*b*). Immunohistological patterns of carcinoembryonic antigen in colorectal carcinoma. Correlation with staging and blood levels. *Pathology,* **12,** 7.

RITZ, J., PESANDO, J.M., NOTIS-MCCONARTY, J., LAZARUS, H. & SCHLOSSMAN, S.F. (1980). A monoclonal antibody to human acute lymphoblastic leukaemia antigen. *Nature,* **283,** 583.

SCHLOM, J., WUNDERLICH, D. & TERAMOTO, V.A. (1980). Generation of human monoclonal antibodies reactive with human mammary carcinoma cells. *Proc. Natl Acad. Sci.,* **77,** 6841.

SIKORA, K. & PHILLIPS, J. (1981). Human monoclonal antibodies to glioma cells. *Br. J. Cancer,* **43,** 105.

SIKORA, K. & WRIGHT, R. (1981). Human monoclonal antibodies to lung-cancer antigens. *Br. J. Cancer,* **43,** 696.

THOMPSON, C.H., JONES, S.L., WHITEHEAD, R.H. & MCKENZIE, I.F.C. (1983). A human breast associated antigen detected by a monoclonal antibody. *J. Natl Cancer Inst.* In press.

UEDA, R., OGATA, S-I., MORRISSEY, D.M. & 6 others. (1981). Cell surface antigens of human renal cancer defined by mouse monoclonal antibodies: Identification of tissue-specific kidney glycoproteins. *Proc. Natl Acad. Sci.,* **78,** 5122.

WHEATER, P.R., BURKITT, H.G. & DANIELS, V.G. (1979). *Functional histology. A text and colour atlas* Edinburgh: Churchill Livingstone p. 253.

YEA, M.Y., HELLSTROM, I., BROWN, J.P., WARNER, G.A., HANSEN, J.A. & HELLSTROM, K.E. (1979). Cell surface antigen of human melanoma identified by monoclonal antibody. *Proc. Natl Acad. Sci.,* **76,** 2927.

ZAMCHECK, N. (1981). The expanding field of colorectal cancer markers: CEA, the prototype. *Cancer Bull.,* **33,** 141.